# Paraneoplastic Limbic Encephalitis Case Report In A Patient With Suspected Conversion Disorder

**DOI:** 10.1192/j.eurpsy.2022.377

**Published:** 2022-09-01

**Authors:** A.N. Duran Öztürk, O. Sahmelikoglu Onur, N. Karamustafalioglu

**Affiliations:** Bakirkoy Research & Training Hospital for Psychiatry, Neurology and Neurosurgery, Psychiatry, Istanbul, Turkey

**Keywords:** paraneoplastic, Conversion Disorder, autoimmune, limbic encephalitis

## Abstract

**Introduction:**

. Autoimmune encephalitis is a difficult-to-recognize, complex disease that can present with various neuropsychiatric symptoms. N-methyl-D-aspartate receptor (NMDA-r) and anti-leucine-rich glioma-inactivated 1 protein (LGI-1) subtypes of autoimmune encephalitis may present with psychiatric symptoms.

**Objectives:**

We would like to present an autoimmune encephalitis case that can be confused with conversion disorder.

**Methods:**

A 54-year-old, female patient started to have forgetfulness ten months ago, and convulsions started five months ago. The patient had disorganized behaviors and contractions in the extremities. Diffusion MRI and brain CT images were normal. The patient had low blood sodium level. In the follow-up, her orientation was impaired and she could hardly make eye contact. As the patient’s contractions were evaluated as conversion in the first stage, 50mg/day sertraline was added to the treatment.

**Results:**

After cranial MRI and EEG recordings were completed, the patient was referred to the neurology department due to the suspicion of autoimmune encephalitis. In the cerebrospinal fluid examination anti-LGI-1 and anti-yo antibodies were positive. Thereupon, IV pulse steroid was given. After that her orientation and disorganized behavior improved. Then, the patient was referred to oncology department.

**Conclusions:**

Limbic encephalitis may manifest as sleep disorders, short-term memory loss, conversion disorder, disorganized behaviors, slurred speech, non-epileptic seizures, sensory and motor defects. Delay in diagnosis may worsen the prognosis of possible malignancy. It should be kept in mind that the patient with a suspected conversion disorder may have limbic encephalitis.

**Disclosure:**

No significant relationships.

